# The Effort and Reward of Teaching Medical Psychology in Germany: an Online Survey

**DOI:** 10.3205/zma001075

**Published:** 2016-11-15

**Authors:** Friederike Kendel, Katrin Rockenbauch, Rolf Deubner, Swetlana Philipp, Götz Fabry

**Affiliations:** 1 Charité – Universitätsmedizin Berlin, Center for Human and Health Sciences, Institute for Medical Psychology, Berlin, Germany; 2 Universitätsklinikum Leipzig, Institute for Medical Psychology and Medical Sociology, Leipzig, Germany; 3 Justus-Liebig-Universität Gießen, Institute for Medical Psychology, Gießen, Germany; 4 Friedrich-Schiller-Universität Jena, Institute for Psychosocial Medicine und Psychotherapy, Jena, Germany; 5 Albert-Ludwigs-Universität Freiburg, Institute for Medical Psychology, Freiburg, Germany

**Keywords:** medical psychology, teaching, effort-reward-imbalance, stress

## Abstract

**Background: **The increasing significance of university teaching also leads to higher demands for academic teachers. Against this background this study inquires how teachers in the field of medical pychology experience and evaluate their various activities and how their efforts on the one hand and gratifications on the other hand relate to each other (as conceptualized by the effort-reward-imbalance, ERI).

**Methods: **A cross-sectional online survey was conducted in 2012 among the academic staff of departments of medical psychology in Germany. The questionnaire was answered by 188 participants (return rate: 39.2%), of whom 62% were women. Work stress was measured according to Siegrist’s effort–reward-imbalance (ERI) model. Further questions referred to the distribution of academic activities and meaningfulness.

**Results: **Among all participants, 67.3% were satisfied with the portion of their workload devoted to teaching, while 63% wanted more time for research. The ERI-coefficient was on average M=0.76 (SD=0.45), thus indicating a shift towards reward. There were no associations with gender, age, or fixed-term work contracts. Meaningfulness was associated negatively with the ERI (r=-.21, p=.012), and positively with overcommitment (r=.52, p<.001) and the desire for less administrative tasks (r=.24, p=.017).

**Conclusions: **Teaching medical psychology is evaluated as positive and meaningful by a majority of respondents. In general, the rewarding aspects seem to outweigh the stressful factors. Thus, teaching might be a protective factor with regard to coping with work related burden.

## Zusammenfassung

**Zielsetzung:** Mit dem zunehmenden Stellenwert der universitären Lehre wachsen auch die Anforderungen an die Lehrenden. Vor diesem Hintergrund wird in dieser Studie untersucht, wie Lehrende im Fach Medizinische Psychologie ihre Tätigkeit erleben und bewerten und in welchem Verhältnis bei ihnen Anstrengungen auf der einen und Gratifikationen auf der anderen Seite stehen (im Sinne der Effort-Reward-Imbalance, ERI).

**Methodik: **Im Jahr 2012 wurde eine querschnittliche Online-Befragung mit Mitarbeitern der medizinpsychologischen Institute in Deutschland durchgeführt. Der Fragebogen wurde von 188 Teilnehmern (62% Frauen) beantwortet (Rücklaufquote 39.2%) und enthielt neben Fragen zur Lehrsituation den ERI-Fragebogen ERI-Q und Items zur Sinnhaftigkeit und Aufteilung der Arbeitsbereiche.

**Ergebnisse:** Von den Befragten waren 67.3% mit dem Anteil der Lehre an ihren Arbeitsaufgaben zufrieden. Für den Forschungsbereich wünschten sich jedoch 63% der Befragten mehr Zeit. Der ERI-Koeffizient lag bei durchschnittlich M=.76 (SD=.45) und damit in einem Bereich, der eine Verschiebung zugunsten der Belohnung anzeigt. Es ergaben sich keine Assoziationen zwischen Geschlecht, Alter oder einer Befristung des Arbeitsverhältnisses mit der ERI. Die ERI korrelierte negativ mit dem Erleben von Sinnhaftigkeit in der Lehre (r=-.21, p=.012) und positiv mit Verausgabungsbereitschaft (r=.52, p<.001) und dem Wunsch nach weniger administrativen Aufgaben (r=.24, p=.017).

**Schlussfolgerung: **Die Lehrtätigkeit in der Medizinischen Psychologie wird von der überwiegenden Mehrheit der Befragten als positiv und sinnhaft beurteilt. Trotz einzelner Belastungsfaktoren scheinen insgesamt die belohnenden Aspekte zu überwiegen. Die Lehrtätigkeit könnte somit ein protektiver Faktor im Hinblick auf den Umgang mit berufsbedingten Belastungen sein.

## Introduction

Academic work promises a high degree of autonomy and prestige and thus also work satisfaction. However, the working conditions in higher education have been more intensively discussed in recent years. This discussion revolves around the (limited) career perspectives of young academics on the one hand and on the increasing demands in research and teaching on the other hand [1]. In research, the pressure is increasing through an ever stronger orientation on acquiring extramural funding and the quantifiable impact of publications [2]. In connection with the Bologna Process or with the general discussion about a stronger orientation toward competency, reforms in teaching have been enacted that change the curricula on a broader basis [3]. These reforms put high demands on the teachers. On the one hand, they are expected to train themselves more intensively in the actual pedagogical aspects of teaching than has ever previously been expected [4]. On the other hand, there are also greater demands on their time, due to the introduction of new forms of teaching and learning, which often involve smaller groups of students and require more intensive preoccupation with the students [5].

Many academic staff take on comprehensive responsibilities in teaching, which, in contrast to research, are often not time-limited projects (i.e. they are activities that will be performed at the school indefinitely). Nonetheless, according to a survey in 2010, only 54% of young academics working at universities had a full-time position, while 42% had only part-time contracts [6]. According to this survey, the average duration of these work contracts was 27 months. Compared to other countries, there are fewer permanent positions beneath the level of a full professor in Germany [6]. In Germany, as in other countries, the financing of researchers is increasingly through project-specific and thus temporary extramural funding, while teaching is increasingly being covered by adjuncts and other freelancers who are paid by the hour. The insecurity resulting from such work conditions and the difficulty of planning a career can impair the satisfaction obtained from work [7]. Moreover, there are indications that the work of academic staff is associated with health complaints, especially stress-related problems, more often than for other occupational groups [8].

### The imbalance of effort and reward

**Figure 1:**
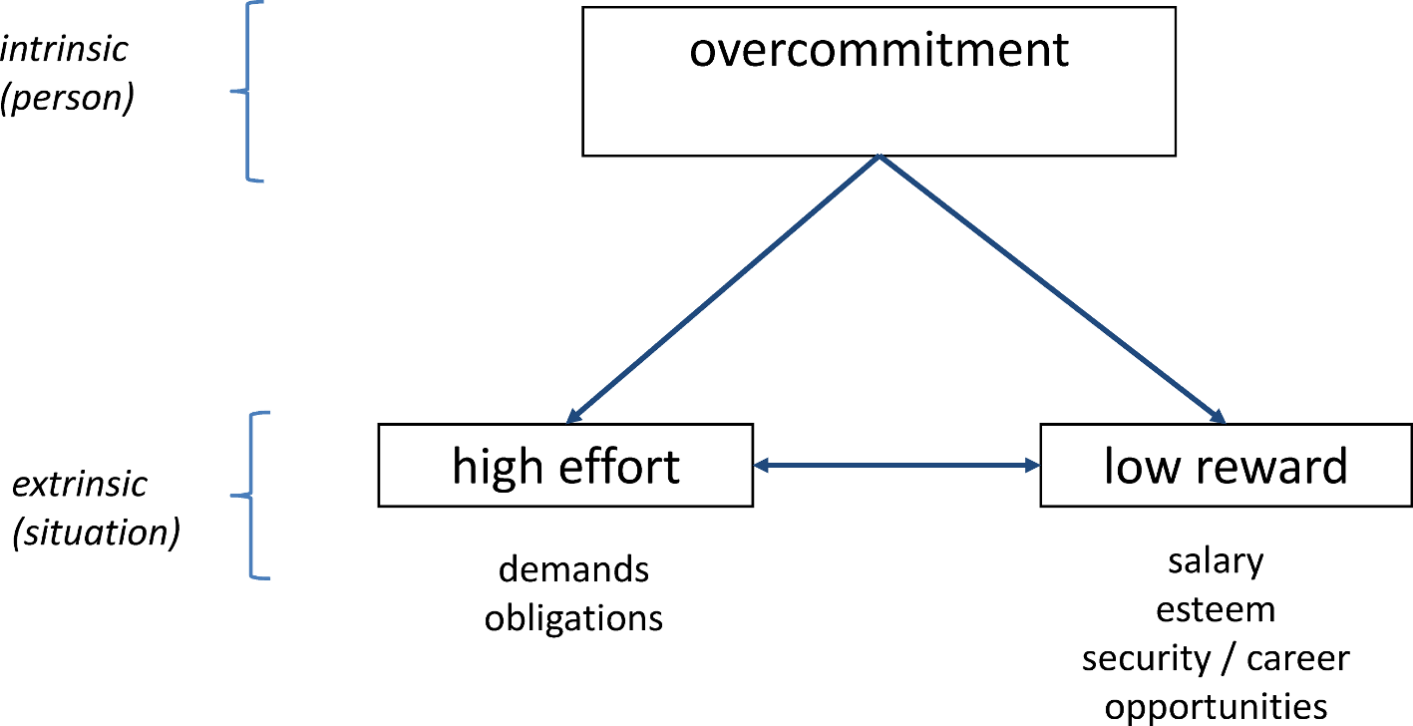
The ERI-Model: Relationship between Effort, Reward and Overcommitment (according to Siegrist 1996) [10].

The complexity of work-related health is reduced to its relevant components with various models [9]. The model of effort/reward imbalance (ERI) [10] is based on the assumption that occupational satisfaction and its effects on health are dependent on the relation between effort and reward. Effort relates to the occupational demands and responsibilities confronting the person. Reward, on the other hand represents extrinsic influence factors that depend above all on the employer, such as pay, appreciation, and the terms of employment. The ERI model postulates an imbalance when the work is characterized by high demands and effort on the one hand but little reward on the other hand. A further component that intensifies this imbalance is overcommitment. People with a tendency to overcommitment react more strongly to an existing imbalance between effort and reward see figure 1 (Fig. 1)). 

An English study on university instructors confirmed these relations [8]. Overcommitment was a strong predictor for anxiety and depression, while reward was associated with a high level of work-satisfaction. Those findings were consistent with the results of an earlier study by Kinman et al. [11]. These authors also showed that an imbalance between effort and reward led to a bad work/life balance, especially for employees who reported increased commitment at work.

#### Medical Psychology in Medicine

There has been an intensive reform process also in medical education during the past 20 years, which has especially affected basic subjects such as medical psychology. A stronger orientation toward practice and a more intensive interrelating of basic sciences and clinical applications has been demanded. The required subject area of medical psychology transmits the basic psychological principles for doctor-patient communication and the methods of social sciences. In addition, it also teaches human development, personality, emotion, and motivation, each with various clinical relations. In research, the departments of medical psychology each have their various foci. In addition to their responsibilities in teaching and research, medical psychologists are often also involved in patient care at the university hospital. The structure and size of departments or divisions of medical psychology is quite variable, yet many departments have a relatively small amount of personnel for the extensive amount of teaching responsibility they are assigned. Despite the fact that medical psychology and medical sociology are listed as one united discipline in the German medical licensure act, the institutionalization of the two disciplines as well as the organization and outline of teaching and the professional interests of the personell can be quite different. Thus, our study focused on the field of medical psychology only.

Against the background of the increasing demands in teaching and research on the one hand and the unreliable career outlook in many cases on the other hand, this study investigates how teachers in the field of medical psychology perceive their employment, and whether they have an imbalance between effort and reward in the sense of the ERI model.

## Methods

### Procedure

The study was initiated by the Teaching Committee of the German Society of Medical Psychology (DGMP). In July 2012, 480 academic staff and professors at all departments of medical psychology in Germany were contacted by email and requested to participate in an online study. 

#### Measurement

The online questionnaire included sociodemographic variables, questions about professional qualifications, teaching experience, current teaching responsibilities, and long-term professional goals. Furthermore, there were five questions about the meaningfulness of the teaching (e.g. my teaching activity advances my own interests” or “I experience my subject field as meaningful”) that were evaluated with a visual analogue scale (0-100). The average of these five items was taken as a unified variable, “the meaningfulness of the teaching” (Cronbach’s α=0.71). The respondents were requested to indicate the percentage division of their own work time among the areas of teaching, research, patient care, and administration. They were also asked to indicate both how much time they actually devote to each of these areas (the “Is” state) and their ideal conception of the time distribution (the “Should Be” state). When the difference between the “Is” and “Should Be” answers was 0-10% (in either direction, positive or negative), a fit between “Is” and “Should Be”, and thus satisfaction with the allocation of time for that activity area, was assumed. A discrepancy of more than 10% was taken as dissatisfaction with the amount of time allocation. 

#### Effort/Reward Imbalance

The Effort/Reward Imbalance Questionnaire (ERI-Q) by Siegrist was used to assess the balance or imbalance between occupational effort and reward [12]. The ERI-Q is a standardized measuring instrument and contains three components. Effort is assessed with five items (a sixth item about physical exertion in the original version is not used for academic occupations). Reward is assessed with 11 items, among which, two refer to appreciation, four to support and financial aspects, and two to employment security. The answers for each scale are summed and the relation between the effort and reward (ERI ratio) is calculated, (multiplying reward by 0.454545, in order to account for the different number of items on the two scales). Higher values on the ERI scale represent an imbalance between effort and reward in the direction of greater effort. The third component in Siegrist’s model is overcommittment, which is assessed with six items.

#### Statistical Analysis

The statistical analysis was performed with SPSS 20.0. To approximate the ERI-coefficient towards a Gaussian distribution, a square root transformation was applied. The t-test for independent samples was used to assess differences between men and women. Pearson’s correlation coefficient was used to ascertain relationships between respondent characteristics and the effort/reward ratio. The statistical significance level was set at α<.05.

## Results

A total of 188 participants (62% women) responded to the online questionnaire, for a response rate of 39.2%. The mean (SD) age was 39.4 (10.5), yet the men were on average 6 years older than the women (see table 1 (Tab. 1)). The respondents’ education was psychology for the majority (66.5%), followed by sociology (18.6%), and medicine (4.3%). Although there was no gender difference regarding the academic discipline, men and women did differ in terms of the level of their education: more women (54.3%) than men (37.3%) had only a bachelor’s or master’s level degree, while more men (15%) than women (7.8%) had completed the habilitation (roughly equivalent to a qualification for tenure-level positions) (p=.003). On average, the respondents had been employed in the field of medical psychology for 7 years, and 75% of the respondents had a time-limited position (62.5% of men, and 82.8% of women, p=.003). Both the gender difference in relation to the level of educational qualification and also the higher portion of men in permanent positions corresponded to a higher portion of men in a leading position (30% vs. 9.2%, p<.001). Regarding the personal long-term goal being pursued, 59.1% of the respondents were working toward a university career. The other 40% of the respondents sought employment in non-university scientific institutions (9.7%), non-university clinical settings (18.8%), or were pursuing other goals (12.4%). The mean (SD) teaching load was 5 (4.02) hours per week during the semester, whereby men taught on average 1.5 such hours more than women (p<.001). A total of 38% of the respondents taught more than 4 hours per week during the semester, while 28.5% of the respondents taught more than was stipulated in their contract. There was no gender difference for that. The mean (SD) score for the “meaningfulness of teaching” scale was 73.2 (17.54). Only 10% of the respondents gave a score < 50.

There were 164 respondents (87.2%) involved in teaching, 157 respondents involved in research (83.5%), 97 respondents (51.6%) involved in administration, and 52 respondents (27.7%) involved in patient care.

**Figure 2:**
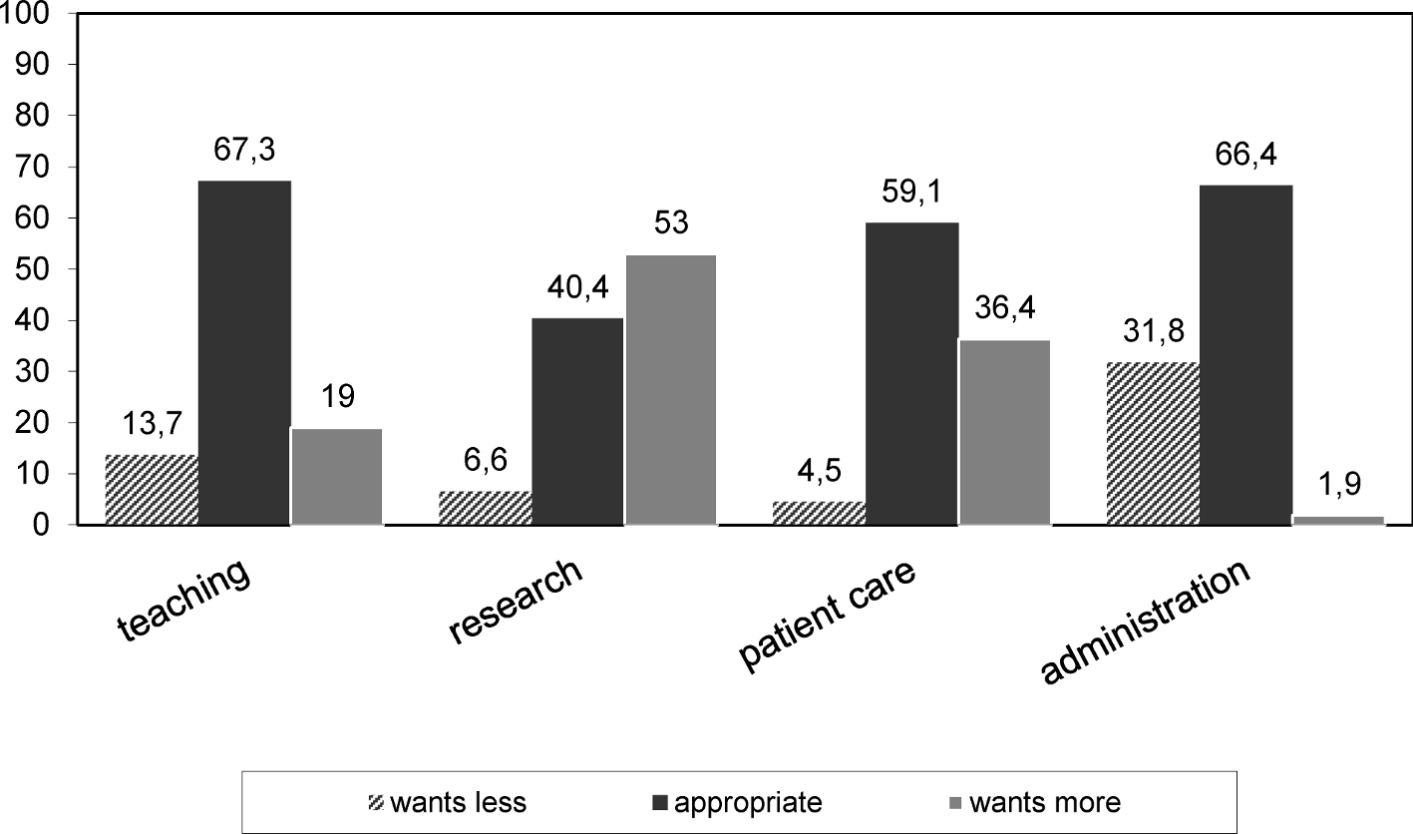
The balance of Teaching, Research, Patient Care and Administration in Medical Psychology (%).

The participants were asked to estimate how much time was spent on the various types of work (“Is State”, see table 2 (Tab. 2)). About one-third of the work time of men (27%) and women (32%) was attributed to teaching, 46% (men) or 42% (women) for research, and 20% for both sexes for administration. Among respondents who take care of patients among their activities, this area was 11% of the work-time of men and 19% of the work-time of women. In addition to an estimation of the “Is State”, the participants were asked about an ideal activity distribution (“Should Be State”, see figure 2 (Fig. 2)). For teaching, 67.3% of the respondents indicated a balanced distribution. Only 13.7% of the participants wanted to spend less time on teaching. Nonetheless, 63% of the respondents wanted more time for research, and only 2% of the participants wanted more time for administrative tasks. Among those doing patient care, only a little more than a third felt that a greater portion of their work-time should be allocated to this area.

### Effort/Reward Imbalance and Overcommitment

Table 3 (Tab. 3) shows the results for effort, reward, and overcommitment. There were no gender differences for any of these scales. The results on the reward scale were on average higher than the results on the effort scale (after adjusting for the different number of items on the two scales). The ERI coefficient had a mean (SD) of 0.76 (0.45) and a range of 0.20 to 2.64. Participants of the survey with an ERI significantly below 1 were in a range that indicated a shift of the balance in favor of reward. 

Table 4 (Tab. 4) shows that sociodemographic variables such as gender, age, or partner status were not associated with the ERI. In contrast, teaching (perceived as being less meaningful), higher overcommitment, fixed-term work contracts, and the desire to spend less time on administrative tasks were significantly associated with a stronger ERI, i.e. a shift towards effort. 

## Discussion

The results of the questionnaire suggest that the teachers at departments of medical psychology in German clearly perceive a favorable relation between effort and reward: The perceived recognition outweighs the reported effort. When interpreting this result, it must be kept in mind, that medical psychology is a theoretical basic subject in medicine. Teaching and research, not patient care, are the primary responsibilities. Teaching in medical psychology requires familiarity with very heterogeneous thematic areas of psychology, and furthermore, multifaceted clinical relevance must be shown. Thus, it is plausible that the question about the meaningfulness of teaching, which also includes the aspect of personal development, was positively answered by the overwhelming majority of respondents. The meaningfulness of teaching was also related with favorable values on the ERI scale, thus with a preponderance of reward. Thus, it appears to be the case that for the teaching activities in medical psychology, there is a good amount of freedom, independence, and possible delivery forms, even under the current university framework conditions. This viewpoint is supported also by the satisfaction with the distribution of time among the various work-areas. It is noteworthy that the wish for a reduction of time is smallest in the area of teaching, even though the respondents had a relatively high amount of teaching activity. Only 14% of the respondents indicated that they wanted to teach less. In contrast, the differences between “Is” and “Should Be” was clearly more pronounced in other areas, e.g. more than half of the respondents wanted more time for research. While about 80% of the respondents experience their work as more rewarding than burdensome, the ERI values of about 20% of the respondents pointed in the other direction: the reported efforts were greater than the perceived rewards or gratification, a result, which was also found in a study with 949 German teachers [13]. The fact that these individuals also experienced less meaningfulness of their teaching activity supports the importance of this construct for satisfaction in the occupational field studied here. The higher overcommitment in this group, by contrast, can be understood in terms of the connection between effort and reward as postulated in the ERI model. The rewarding aspects of their activity apparently cannot outweigh the invested efforts. On the basis of our data it cannot be decided whether the imbalance in this group regarding the perceived meaningfulness as well as the overcommitment can be attributed more to individual predispositions or to the surrounding conditions. On the one hand, teaching may be perceived as less meaningful or a person may have an inherently greater overcommitment. On the other hand, our results point to the fact that too great a portion of time for administrative tasks can also unfavorably influence the ERI. Further unfavorable surrounding conditions could be strict curricula that may limit the sense of achievement. 

It was surprising that the ERI results were not higher among employees with temporary work-contracts and less teaching experience. Thus the supposition that employment conditions characterized by an insecure future are accompanied principally by a higher risk for burnout [14] was not supported by our results. Perhaps the aspect of personal development is more important here than the strain that can be associated with an insecure employment condition. Variables that have been shown to be relevant in other studies in the medical context, e.g. “unfavorable organizational work conditions in the hospital” or “overwork through lack of sleep” [15] are surely less pronounced in our target population. Although about one third of the employees of medical psychology are also active in patient care, this activity is not normally their main focus. Thus, medical psychology may be compared better to social or natural science fields, in which, despite widely existing complaints about stress and a high workload of research and teaching, apparently many academics show a high intrinsic motivation and identify strongly with their activities [16], [17]. The degree to which the introduction of modular study programs decreases the scope for how to design the teaching and thereby also the work remains to be seen. satisfaction remains to be seen [18].

Although substantially more male than female respondents were in a leading position, there were no gender differences for the balance of recognition and exertion. Aster-Schenk and colleagues showed [19] that even male medical students are still more ambitious regarding a professional career while female students define job-related success more in terms of a succesful balance of work and family life. This is also supported by Hohner et al. [20] who interviewed male and female physicians and concluded that „especially for women an alternative definition of job-related success with a broader focus becomes more important as a guideline. In this light a succesful career trajectory is often defined as one that integrates ideally with the private way of life.“ This could mean that individual components of the model of occupational gratification crises are appraised differently by men and women. If salary and status are less important and positive teaching experiences weigh more in the scales, then women could perceive their work as rewarding, despite a comparatively lower salary and less status. A further explanation could be the particularity of the social networks, in which women receive more social support in a predominantly female field such as medical psychology than in other subject areas of the medical university. In a study with academic personnel, women benefited from a supportive departmental atmosphere in particular regarding their emotional exhaustion [21], and in general, a stronger social support is associated with less occupational stress and burnout [22].

## Limitations

Our study has several limitations.

1. The response rate was only about 40%. Yet this is a level corresponding to other online surveys. The gender ratio (62% women), the ratio of temporary versus permanent staff, and the portion of participants in leading positions speak for the representativeness of the sample. Nevertheless, we cannot rule out that our sample is biased since more burdened participants did not complete the survey more frequently.[23];
2. A further limitation concerns the design of a cross-sectional study. Causal interpretations or statements about development are not possible. A longitudinal study design with repeated measures, which could measure changes over time and the influence of gratification crises on mental and physical health, would be desirable [15]. Capturing additional variables such as depressiveness or perceived social support at the workplace would be desirable.

## Conclusions

Work in the field of medical psychology is viewed as positive and meaningful by the majority of respondents. Longitudinal studies should be drawn upon, in order to show the degree to which this appraisal is stable over time also for temporary work contracts, and to determine how an unfavorable relation between expenditure and reward influences mental and physical strain.

## Acknowledgements

The authors would like thank the Directors of the German Society of Medical Psychology for the support during the planning and carrying out of the survey. We would also like to thank Michael Hanna, PhD, for translating the manuscript from German into English.

We dedicate this article in loving memory to our colleague Wolfgang Hannöver who untimely passed away. We miss him very much.

## Competing interests

The authors declare that they have no competing interests.

## Figures and Tables

**Table 1:**
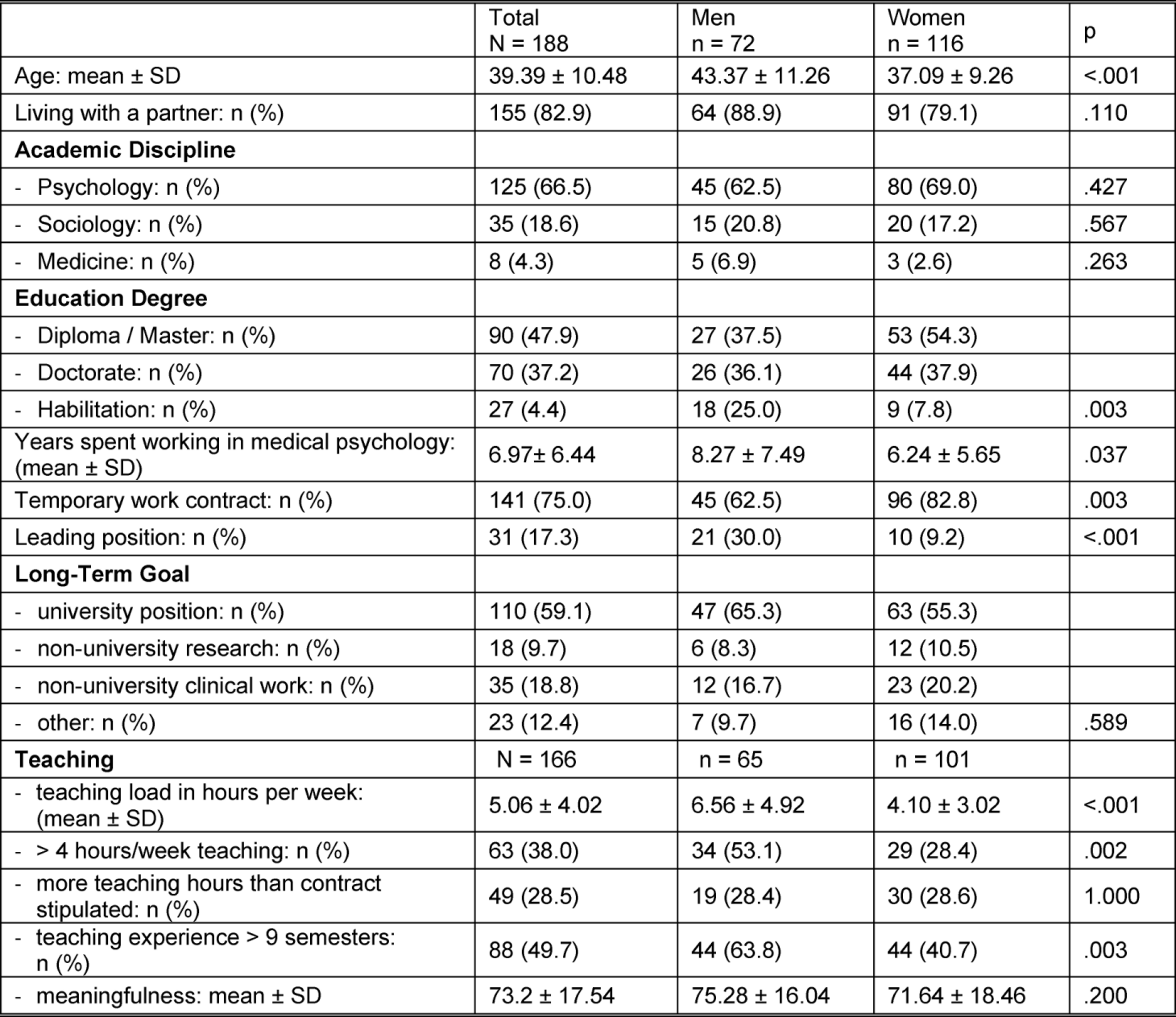
Descriptive Statistics and Gender Comparison

**Table 2:**
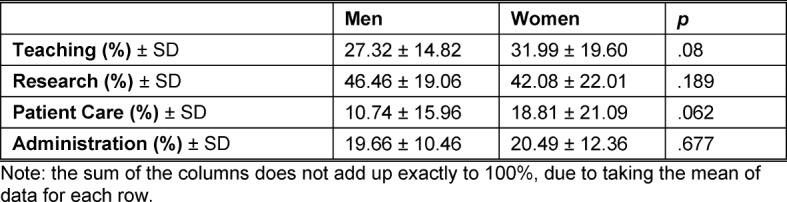
Percent Distribution of Time among the Various Work Areas, compared by gender.

**Table 3:**
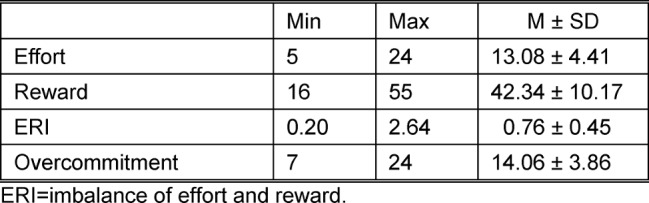
ERI and Overcommitment

**Table 4:**
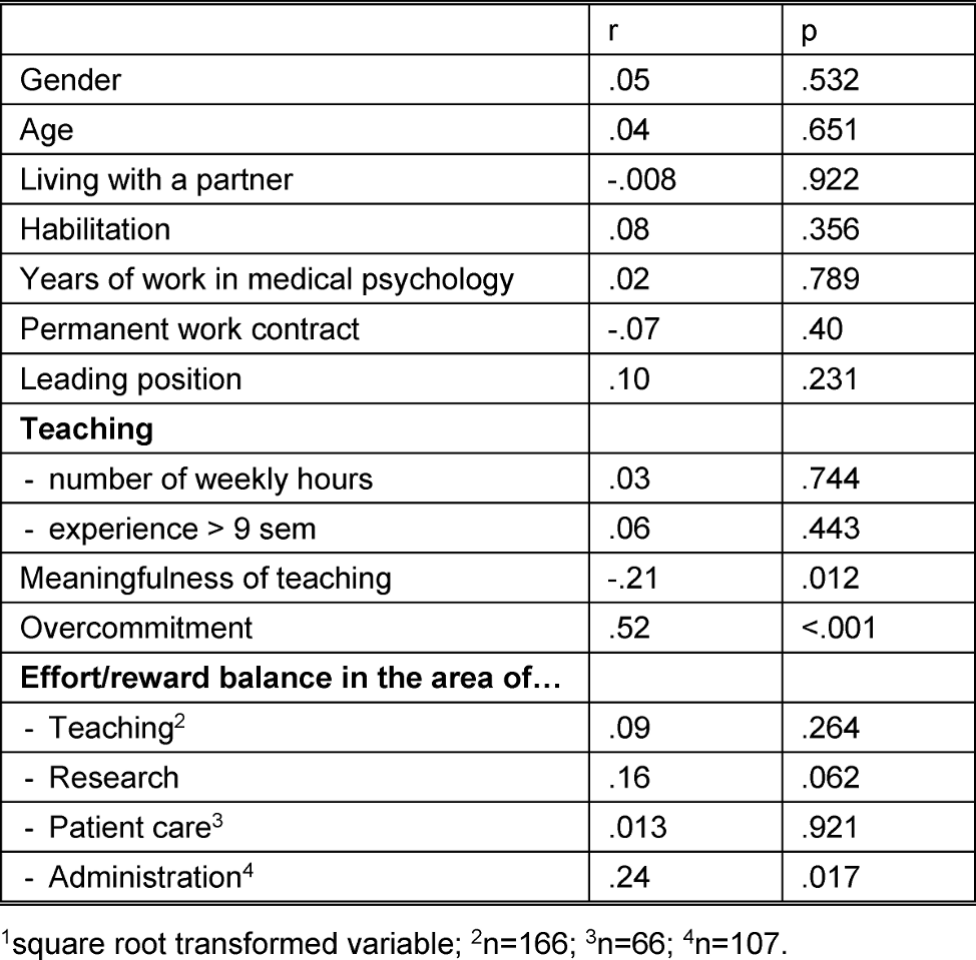
Correlations of Selected Variables with the Effort/Reward Imbalance^1^
